# Prevalence and Associated Factors of Full Immunization Coverage among children aged 12–23 months in Kenya: A multi-level logistic regression analysis

**DOI:** 10.1371/journal.pone.0361187

**Published:** 2026-11-06

**Authors:** Gebeyehu Lakew, Demis Getachew, Eyob Getachew, Abiyu Abadi Tareke, Amlaku Nigusie Yirsaw

**Affiliations:** 1 Department of Health Promotion and Health Behavior, Institute of Public Health, College of Medicine and Health Sciences, University of Gondar, Gondar, Ethiopia; 2 Department of Pharmacology, School of Pharmacy, College of Medicine and Health Sciences, University of Gondar, Gondar, Ethiopia; 3 Zonal-Level COVAX and Routine Immunization Technical Assistance (TA) at West Gondar Zonal Health Department, Gendawuha, Ethiopia; King Caesar University, UGANDA

## Abstract

**Background:**

Full immunization coverage remains suboptimal in Kenya, despite the availability of effective vaccines. Understanding the multilevel factors influencing immunization uptake is essential for strengthening child health interventions. This study examines individual and community-level determinants of full immunization coverage among children aged 12–23 months in Kenya.

**Objective:**

To assess the individual and community-level factors associated with full immunization coverage among children aged from 12–23 months in Kenya using a multilevel analytical approach.

**Methods:**

A cross-sectional study was conducted using nationally representative survey data from Kenya. A two-level binary logistic regression model was used to identify individual- and community-level factors associated with full immunization coverage in Kenya. Adjusted odds ratios (AOR) with 95% confidence intervals (CI) were reported.

**Results:**

The prevalence of full immunization coverage among children aged from 12–23 months in Kenya was 79.4% (95% CI: 78.13–81.25). A total of 3790 weighted children aged from 12–23 months were included. Children whose mothers were exposed to media (AOR = 1.16, 95% CI: 1.01–1.34), delivered in health facilities (AOR = 2.88, 95% CI: 2.43–3.42), and attended antenatal care (AOR = 3.15, 95% CI: 2.10–4.73) had higher odds of full immunization. Children of average birth size (AOR = 1.68, 95% CI: 1.40–2.01) and those from rural areas (AOR = 1.44, 95% CI: 1.22–1.69) were also more likely to be fully immunized. Higher maternal education showed a strong positive association: primary (AOR = 1.60), secondary (AOR = 2.14), and higher education (AOR = 1.74). Older maternal age increased immunization odds: 25–34 years (AOR = 1.49), 35–49 years (AOR = 2.15). Conversely, households with more than two under-five children (AOR = 0.72) and higher parity (2–5 children AOR = 0.78; 6 + AOR = 0.67) had lower odds. Community-level low maternal education also reduced the odds (AOR = 0.79).

**Conclusion and recommendation:**

Maternal age, education, parity, media exposure, antenatal care, place of delivery, place of residence, and community education levels were associated with full immunization coverage in Kenya. Interventions should focus on improving maternal education, strengthening ANC services, promoting institutional deliveries, and enhancing outreach through media and community engagement.

## Background

Immunization has played a vital role in global public health by preventing millions of deaths and reducing the burden of vaccine-preventable diseases, especially among children under five years of age [1]. The World Health Organization (WHO) estimates that immunization currently prevents 4–5 million deaths annually and could prevent even more with improved coverage [2]. Despite this progress, disparities in vaccination coverage remain, particularly in low- and middle-income countries where systemic barriers often limit access to routine immunization services [3].

Sub-Saharan Africa continues to report some of the lowest immunization rates globally, contributing significantly to the global burden of vaccine-preventable illnesses [2]. Factors such as limited maternal education, lack of healthcare access, socioeconomic constraints, and cultural beliefs have all been identified as key contributors to under-vaccination in this region [4].

In Kenya, immunization is delivered through the National Vaccines and Immunization Programme (NVIP**),** aiming to ensure that every child receives the basic immunization schedule by their first birthday. Although the country has made commendable efforts in **National Vaccines and Immunization Programme** recent data show that full immunization coverage among children aged 12–23 months remains at 68.8%, falling short of the national target of 90% and the global target of 95% [5]. This gap underscores the importance of understanding individual and contextual barriers to immunization uptake within Kenyan settings.

While some studies have explored the predictors of vaccination in Kenya, few have employed multilevel approaches that account for the influence of individual and community-level determinants. Such a comprehensive analysis is crucial for developing targeted and effective strategies to improve vaccine coverage and equity in the country [6].

This new study looks at community-level and individual-level factors that contribute to childhood full immunization in Kenya to provide a more thorough understanding [7,8].

Multivariable multilevel logistic analysis, a more sophisticated method that accounts for data clustering, is used in this new study to address this problem. It enhances earlier study techniques that did not adequately account for how children are grouped within communities. The study offers a more accurate interpretation by employing a more robust statistical technique.

In the end, a two-level binary logistic regression model was used to identify individual- and community-level factors associated with full immunization coverage in Kenya.

## Methods

### Study area, data source, and study period

Between February 17, 2022, and July 13, 2022, the Kenya Demographic and Health Survey (KDHS) 2022 was carried out. This survey offers thorough information on nutrition, health, and demographic factors throughout Kenya, making it an essential tool for research, program implementation, and policy-making in the nation's health sector.

### Sampling technique and study population

The Enumeration zones created during the 2022 Kenya Population and Housing Census served as the basic sample frame for the KDHS's well-structured sampling strategy. By using the comprehensive, up-to-date data collected during the national census, this method ensures the representativeness of the survey results.

The comprehensive sampling design of the 2022 Kenya Demographic and Health Survey (KDHS) ensures a nationally representative dataset covering both urban and rural areas across all 47 counties. The study selected 1,692 clusters (enumeration areas), including 666 urban and 1,026 rural clusters, and systematically sampled 25 households per cluster, resulting in a total sample of 42,300 households. Among these, 37,911 households were successfully interviewed, ensuring a high response rate. To assess full immunization coverage among children aged from 12–23 months, Information on children's vaccination status was obtained from mothers or caregivers, who were asked whether the child had received the recommended basic vaccinations before the survey.

In the context of the study at hand, the resulting sample size comprises 3,790(weighted) children aged from 12–23 months. The KDHS 2022 employed a two-stage stratified cluster sampling design. To obtain nationally representative estimates, the DHS individual sampling weights (v005) were applied after dividing by 1,000,000, following the KDHS 2022 methodology. The weights account for unequal probabilities of selection and non-response. For descriptive analyses, the survey design was specified using the primary sampling units and sampling strata. For the multilevel logistic regression analysis, individual sampling weights were incorporated as probability weights while accounting for clustering at the community level. This careful consideration of the sampling strategy and the subsequent weighting of the sample enhance the validity and reliability of the findings, providing valuable insights into the health and demographic dynamics of children aged from 12–23 months in Kenya as of 2022.

#### Dependent variable.

The dependent variable in this study was full immunization coverage. For the multilevel analysis, immunization coverage was dichotomized such that children who were not immunized or only partially immunized were coded as ‘0’ (not fully immunized), and those who received all recommended vaccines by the age of 12–23 months were coded as ‘1’ (fully immunized).

#### Independent variables.

The independent variables were selected based on a thorough review of existing literature, theoretical relevance, and their availability in the Kenya DHS dataset.

In Kenya, several individual-level factors were examined for association with full immunization coverage, including the child's age and sex, maternal age and education, economic status, maternal working status, the number of children under five in the household, breastfeeding status, place of birth, birth size, antenatal care attendance, the sex of the household head, place of residency, parity and media exposure. Moreover, community-level factors, such as overall educational attainment and poverty within the community, were also considered.

### Operational definition

**Full immunization:** Full immunization was assessed among children aged 12–23 months according to the KDHS 2022 basic vaccination schedule. A child was considered fully immunized if he or she had received one dose of BCG, three doses of polio vaccine (excluding the birth dose), three doses of pentavalent vaccine, three doses of pneumococcal conjugate vaccine (PCV), two doses of rotavirus vaccine, and one dose of a measles-containing vaccine. Vaccination status was determined using vaccination card records where available and maternal/caregiver recall when vaccination cards were unavailable
**Media Exposure:** Newspapers, radio, and television were all combined into one category, “exposed to media,” for this study to guarantee a wider range of participants. If a person reported utilizing any of these media sites, they were placed in this category. This approach helps avoid participant exclusion, which may happen if each medium were examined independently. For instance, educated women may be more likely to read newspapers, but only those with electricity may be able to watch television. These sources are combined to give the study a more thorough analysis.
**Size at birth**: was assessed using the mother's subjective assessment recorded in the KDHS 2022. The original five response categories (very small, smaller than average, average, larger than average, and very large) were recoded into three categories: **small** (very small and smaller than average), **average**, and **large** (larger than average and very large).
**Community Poverty Level**: Community-level poverty was generated by aggregating the proportion of households in the poor and poorer wealth quintiles within each enumeration area. Because the distribution was non-normal, the aggregated variable was categorized into **high community poverty (**≥**45%)** and **low community poverty (<45%)** using the national median value as the cut-off.
**Community level of educational status**: Community-level educational attainment was generated by aggregating the proportion of women with low educational attainment within each enumeration area (cluster). Because the distribution was non-normal, the aggregated variable was categorized into **high community-level low educational attainment (**≥**40%)** and **low community-level low educational attainment (<40%)** using the national median value as the cut-off.

### Data management and analysis

This study used tables, percentages, and medians to show the main features of the data. Excel was used to clean the data by checking for duplicate observations, missing values, out-of-range and inconsistent values, and coding errors. The analysis was restricted to children aged 12–23 months. Variables were recoded according to the KDHS 2022 recode manual, and records with complete information on the outcome variable and selected explanatory variables were included in the final analysis. Weighted frequencies were used to address any disparities in group representation in the sample. The KDHS 2022 employed a two-stage stratified cluster sampling design. To obtain nationally representative estimates, the DHS individual sampling weight (v005) was divided by 1,000,000 and applied following DHS recommendations. The survey design accounted for the primary sampling units (v021) and sampling strata (v022/v023) during the descriptive analyses. All analyses were performed using **Stata version 17.0**.

### Mixed model

This study identified characteristics associated with full immunization coverage in Kenya using a multilevel logistic regression. When evaluating data with nested structures, such as the Demographic and Health Survey (DHS) data, this method is especially helpful.

However, the analysis goes beyond just individual characteristics. It also considers how community-level factors might influence these individual traits. For instance, a mother's education and income might be affected by where she lives (enumeration area), the overall media exposure in her community, and the community's poverty level. By taking these community factors into account, the study can provide a more comprehensive picture of what contributes to childhood full immunization coverage.

The Demographic and Health Survey (DHS), which gathers data at the individual and community levels, provided the data used in this investigation. Children in the same community (enumeration region) are probably more alike than children from other communities, resulting in a nested structure.

This study used a two-level binary logistic regression model to identify factors associated with full immunization coverage in Kenya. This model considers both individual and community-level characteristics. We compared four different models. The first model, called the “null model” or “random intercept model,” didn't include any explanatory variables. It was used to assess how much variation in immunization exists between communities. Model comparison was performed using the deviance (−2 log likelihood), and the model with the lowest deviance was considered the best-fitting model. In other words, this model best explains the factors associated with childhood full immunization coverage.

To assess the variation of full immunization coverage prevalence between communities in Kenya, we used three key measures. The intra-class correlation coefficient (ICC) indicated what proportion of the overall difference in full immunization coverage could be attributed to factors specific to each community. The proportional change in variance (PCV) measured how much this variation between communities decreased after accounting for other factors in the analysis, compared to a model with no explanatory variables. Finally, the median odds ratio (MOR) provided a different perspective. The value of ICCs and MORs was estimated from intercept-only models (null model) to examine the presence of clustering and heterogeneity of full immunization coverage between areas. Finally, variables with p-value < 0.25 were considered for multivariable analysis. This threshold was chosen to avoid excluding potentially important predictors and confounding variables during the initial screening stage. In addition, variables identified as important in previous studies were retained irrespective of their bi-variable statistical significance. An adjusted odds ratio with a 95% confidence interval (CI) and p-value <0.05 were used to declare statistical significance.

## Result

### Characteristics of the study population

Most research participants, 2,524 mothers, or 66.60%, came from rural areas, and 37.95% of these children were born to mothers who had completed basic school (Table 1).

**Table 1:** Demographic features of the study participants (n = 3,790) in Kenya for the year 2022.

Characteristics	Weighted frequency	Percent
**Individual level factors**		
**Sex of the child**		
Male	1,938	51.13
Female	1,852	48.87
**Maternal age**		
15-24 years	1,211	31.95
25-34 years	1,847	48.73
35-49 years	732	19.31
**Place of residence**		
Urban	1,266	33.40
Rural	2,524	66.60
**Maternal educational status**		
No education	809	21.35
Primary school	1,316	34.72
Secondary	1,183	31.21
Higher	482	12.72
**Household’s Wealth status**		
Poorest	1,250	32.98
Poorer	682	17.99
Middle	651	17.18
Richer	685	18.07
Richest	522	13.77
**Mother’s Current working status**		
No	2,124	56.04
Yes	1,666	43.96
**No. of under-five children in the households**		
≤2 children	3,240	85.49
>2children	550	14.51
**Media exposure**		
Exposed	990	26.12
Not exposed	2,800	73.88
**Place of delivery**		
Home	771	20.34
Health facility	3,019	79.66
**Breastfeeding status**		
No	1,182	31.19
Yes	2,608	68.81
**Sex of the household head hold**		
Male	2,681	70.74
Female	1,109	29.26
**Size of child**		
Large	313	8.26
Average	3,101	81.82
Small	376	9.92
**Antenatal care followup**		
No	133	3.51
Yes	3,657	96.49
**Parity**		
1	1,019	26.89
2-5	2,185	57.65
6 and above	586	15.46
Community level factors		
**Community level educational status**		
Community with low level of educational status	2,532	66.81
Community with high level of educational status	1,258	33.19
**Community level of poverty**		
High level of poverty	1,685	44.46
Low level of poverty	2,105	55.54

### Prevalence of full immunization coverage among children aged from 12–23 months in Kenya

The prevalence of full immunization coverage among children aged from 12–23 months in Kenya was 79.4% (95% CI: 78.13–81.25).

#### Model fitness parameters.

Based on the model fitness parameters assessed for full immunization coverage, Model III demonstrated the best overall fit compared to the other models. This conclusion is supported by its lower values across key criteria: the Akaikian Information Criterion (AIC = 13,655.84), Bayesian Information Criterion (BIC = 13,884.21), and Deviance Information Criterion (DIC = 13,597.84), all of which are commonly used to evaluate model performance. Additionally, the log-likelihood ratio (−6798.92) for Model III was the highest among the models, indicating better model likelihood. The null model demonstrated significant between-community variation in full immunization status, with an intra-class correlation coefficient (ICC) of **3.81%,** indicating that approximately 3.8% of the total variation in full immunization was attributable to differences between clusters. After adjusting for individual- and community-level variables, the between-cluster variation changed across the fitted models, with ICC values of **7.71%, 3.44%,** and **7.72%** for Models I, II, and III, respectively. The median odds ratio (MOR) ranged from **1.39** to **1.65**, indicating persistent heterogeneity in the odds of full immunization between clusters after accounting for the measured covariates. Model III explained the greatest proportion of the community-level variation, with a proportional change in variance (PCV) of **11.3%.** Furthermore, Model III had the **lowest deviance (−2 log likelihood) and** Akaike Information Criterion (AIC**),** indicating that it provided the best fit to the data among the four fitted models (Table 2).

**Table 2:** Comprehensive Comparison of Models and Outputs of Fitness Parameters for Assessing full immunization coverage among children aged from 12-23 months in Kenya.

Fitness parameter	Null model	Model I	Model II	Model III
Community level variance	0.130315	0.11698	0.1171929	0.11559
ICC	3.81%	7.71%	3.44%	7.72%
MOR	1.41[1.32,1.69]	1.65[1.48,1.71]	1.39[1.31, 1.46]	1.65 [1.61, 1.71]
PCV	Reference	11%	10.06%	11.3%
**Model fitness parameters**				
Log- likelihood ratio (LLR)	−10740.814	−6803.2439	−10710.553	−6798.9176
DIC(−2LLR)	21,481.628	13,606.4878	21,421.106	13,597.8352
AIC	21485.63	13660.49	17212.4	13655.84
BIC	21501.38	13873.11	17243.9	13884.21

**NOTE**: ICC, intra-cluster correlation; MOR, median odds ratio; DIC, deviance information criterion. The null model is the empty model, the base model without any determinant variable. **Model I** is adjusted for individual-level factors. Model II is adjusted for community-level factors**.** Model III is the final model adjusted for both individual and community-level factors.

### Factors associated with full vaccine coverage among children aged from 12–23 months in Kenya

This multilevel analysis showed key individual and community-level factors associated with full immunization coverage. Mothers who were exposed to media had 1.16 times higher odds of fully immunizing their children compared to those without media exposure. Households with more than two children under the age of five had 28% lower odds of full immunization (OR = 0.72) than those with two or fewer children.

Children born in health facilities had significantly higher odds of being fully immunized (OR = 2.88) compared to those born at home. Children of average birth size were 1.68 times more likely to be fully immunized compared to those with large birth size.

Mothers who attended antenatal care were over three times more likely to fully immunize their children (OR = 3.15) than those who did not. Children from rural areas had 1.44 times higher odds of full immunization than their urban counterparts.

Maternal age was positively associated with full immunization. Mothers aged 25–34 had 1.49 times the odds, and those aged 35–49 had 2.15 times the odds, compared to mothers aged 15–24. Increased parity was linked to lower immunization rates. Mothers with 2–5 children had 22% lower odds (OR = 0.78), and those with six or more children had 33% lower odds (OR = 0.67) than first-time mothers.

Educational status showed a positive gradient with immunization. Compared to mothers with no education, those with primary education had 1.60 times the odds, those with secondary education had 2.14 times the odds, and those with higher education had 1.74 times the odds of fully immunizing their children.

At the community level, children living in communities with low maternal education had 21% lower odds of being fully immunized (OR = 0.79) than those living in communities with higher educational levels (Table 3).

**Table 3:** Multivariable multilevel logistic analysis of full immunization among children aged from 12 - 23 months in Kenya.

Variable Name	AOR	p-value	95% CI
**Media exposure**			
No	1		
Yes	1.16	0.047	1.01 - 1.34
**No. of under-five children in the households**			
≤2 children	1		
>2children	0.72	p < 0.001	0.62− 0 .84
**Place of delivery**			
Home	1		
Health facility	2.88	p < 0.001	2.43- 3.42
**Size of child at birth**			
Large	1		
Average	1.68	p < 0.001	1.40-2.01
Small	1.45	0.091	0.95__2.21
**Antenatal care visit**			
No	1		
Yes	3.15	p < 0.001	2.10- 4.73
**Type of place of residency**			
Urban	1		
Rural	1.44	p < 0.001	1.22-1.69
**Educational status**			
No education	1		
Primary	1.35	0.073	
Secondary education	1.30	0.024	1.03-1.65
Higher	1.24	0.094	0.96-1.37
**Age of mother**			
15-24	1		
25-34	1.49	p < 0.001	1.32-1.69
35-49	2.15	p < 0.001	1.81-2.55
**Parity**			
1	1		
2-5	0.78	p < 0.001	0.69-0.88
6 and above	0.67	p < 0.001	0.54-0.83
**Educational status**			
No education	1		
Primary	1.60	0.002	1.18- 2.17
Secondary	2.14	p < 0.001	1.53- 2.99
Higher	1.74	0.003	1.21- 2.51
**Community level factor**			
level of education			
High	1		
Low	0.79	0.015	0.65- 0.95

## Discussion

The present study revealed that the prevalence of full immunization coverage among children aged from 12–23 months in Kenya was 79.4% (95% CI: 78.13–81.25), which is consistent with prior national estimates and remains below the recommended target of 90% by the World Health Organization [9,10].The multilevel logistic regression analysis identified several individual- and community-level factors significantly associated with full immunization coverage.

One of the associated factors of full immunization was antenatal care (ANC) visits, where mothers who had at least one ANC visit were more than three times more likely to fully immunize their children compared to those who did not. This finding aligns with previous studies in Ethiopia [11] suggesting that ANC visits provide critical opportunities for health education and vaccination counseling.

Similarly, place of delivery was associated with full immunization coverage; children born in health facilities were nearly three times more likely to be fully immunized than those born at home. Institutional deliveries often ensure immediate postnatal care and early vaccination initiation, which lays the foundation for continued immunization [12]. This finding mirrors a multilevel analysis conducted across sub-Saharan Africa, where institutional delivery was significantly associated with increased immunization uptake [13,14].

Maternal age showed a positive association, with older mothers (especially those aged 35–49 years) more likely to fully vaccinate their children compared to younger mothers. Older mothers may have more experience, autonomy, and better access to health information, which enhances healthcare-seeking behavior. A similar pattern has been observed in India. This study indicated that older mothers had a higher likelihood of fully immunizing their children, potentially due to greater experience and access to health resources

Educational attainment of the mother was also another associated factor. Mothers with secondary education were more than twice as likely to fully vaccinate their children compared to those with no education. This is consistent with findings from Ghana [15]. Higher education is frequently associated with improved health literacy and utilization of maternal and child health services [16]. Moreover, at the community level, residing in communities with low educational status was associated with lower odds of full immunization, indicating the influence of broader social and environmental factors.

Media exposure slightly increased the odds of full immunization, highlighting the role of health communication in raising awareness. This is in line with findings from studies in Bangladesh and Uganda where mass media was linked to increased vaccine uptake [17,18].

Regarding household characteristics, having more than two under-five children was negatively associated with full immunization. This may reflect competing demands on caregivers’ time and resources, thereby limiting their ability to adhere to vaccination schedules. Similarly, higher parity (six or more children) was associated with lower immunization coverage, echoing previous studies suggesting that mothers with many children often face increased financial and logistical challenges [19].

Children perceived by their mothers as having an average size at birth had higher odds of being fully immunized than those perceived as large. Although the underlying mechanism cannot be determined from this cross-sectional study, one possible explanation is that caregivers may perceive infants of average size as requiring closer adherence to routine preventive healthcare, including immunization. However, this interpretation is speculative and warrants further investigation in longitudinal or qualitative studies. Contrary to expectations, children residing in rural areas had higher odds of being fully immunized than those living in urban areas. This finding differs from observations reported in some previous studies but is consistent with evidence from other sub-Saharan African settings [20]. One possible explanation is that rural immunization outreach services or targeted vaccination programs may have improved access to childhood immunization in these areas. Alternatively, differences in healthcare utilization, community health worker engagement, or local immunization strategies may have contributed to this finding. Because the present study was cross-sectional, these explanations should be interpreted as hypotheses rather than causal conclusions.

### Implications of the study

The findings of this study highlight several factors that may be relevant to efforts to improve full immunization coverage among children in Kenya. The observed associations between antenatal care attendance, institutional delivery, maternal education, media exposure, household characteristics, and immunization coverage suggest areas that could be considered in immunization planning and health promotion activities. Strengthening opportunities for vaccination counselling during antenatal and facility-based maternal and child health services may be considered as part of broader strategies to support immunization uptake. Similarly, maternal education and appropriate health communication through media and community-based platforms may provide opportunities for improving awareness of childhood immunization.

The observed association between household characteristics and immunization coverage also indicates the importance of considering the practical challenges faced by households with multiple young children when designing immunization services. The higher odds of full immunization observed among children in rural areas may warrant further investigation to understand the potential contribution of outreach services, community health workers, and other context-specific factors.

Because this study used cross-sectional survey data, these findings should be interpreted as associations rather than evidence that specific interventions would directly increase immunization coverage. Further longitudinal, qualitative, and intervention research could help determine whether the identified factors are amenable to specific interventions and which approaches are most effective in different Kenyan settings.

### Strengths and limitations of the study

The study’s strengths include using a two-level binary logistic regression model was used to identify individual- and community-level factors associated with full immunization coverage in Kenya. A large, nationally representative sample increases the generalizability of the findings, and the inclusion of key variables like maternal education and ANC visits provides a comprehensive understanding of immunization coverage. However, several limitations should be considered when interpreting the findings. First, because this study was based on cross-sectional KDHS data, causal relationships between the identified factors and basic vaccination coverage cannot be established. Second, vaccination status was determined using vaccination cards where available and maternal or caregiver recall when cards were unavailable, which may have introduced recall bias and potential misclassification of the outcome. Third, the KDHS provides information on whether vaccines were received but does not allow assessment of the timeliness of vaccination, an important indicator of immunization programme performance. Fourth, although a wide range of individual- and community-level variables were included, residual confounding from unmeasured factors, such as parental attitudes toward vaccination, vaccine availability, healthcare quality, and cultural beliefs, cannot be excluded. Furthermore, this study used nationally representative KDHS survey data and did not analyze routine administrative immunization data from the Kenya Health Information System (KHIS/DHIS2). Therefore, the estimated vaccination coverage reported in this study should not be directly compared with routine administrative coverage estimates because the two data sources differ in their methods of data collection, estimation, and reporting.

## Conclusion

This study identified several individual- and community-level factors associated with full immunization coverage among children aged 12–23 months in Kenya, including antenatal care attendance, place of delivery, maternal education, media exposure, household characteristics, and place of residence. These findings highlight potential areas for consideration in immunization planning and health promotion activities. However, the cross-sectional nature of the study limits causal inference, and the findings should therefore be interpreted as associations rather than demonstrated intervention effects. Further longitudinal, qualitative, and intervention research is needed to better understand the mechanisms underlying these associations and to identify effective approaches for improving childhood immunization coverage.

## Supporting information

S1 File Human participant research checklist for full immunization. (DOCX)

## Abbreviations

AIC: akaike information criteria
ANC: antenatal care
AOR: adjusted odds ratio
BIC: Bayesian information criteria
DIC: deviance information criterion
ICC: intra-class correlation
LLR: log-likelihood ratio
MOR: median odds ratio
PCV: proportion of variance change
CI: confidence interval
AOR: adjusted odds ratio
EA: enumeration areas
KM: kilometer
KDHS: kenya demographic health survey
