# Supplementary material for: Prevalence and Associated Factors of Full Immunization Coverage among children aged 12–23 months in Kenya: A multi-level logistic regression analysis
Source: PLoS One. 2026 Nov 6;21(11):e0361187. doi: 10.1371/journal.pone.0361187 (PMC13742959; doi:10.1371/journal.pone.0361187)
Supplement: Supplementary file 1 [file pone.0361187.s004.pdf]

Response to Reviewer 4

We sincerely thank the reviewer for the careful and constructive evaluation of our manuscript. We appreciate the comments regarding statistical analysis, data availability and ethics, language, and overall presentation. We have carefully reviewed the manuscript and incorporated the requested revisions. The revised manuscript has also undergone comprehensive proofreading to improve clarity, consistency, grammar, terminology, numerical presentation, and formatting.

Comment 1: Statistical Analysis

Reviewer comment:
The statistical approach is appropriate, particularly the use of multilevel logistic regression to account for the hierarchical structure of the DHS data. The manuscript appropriately reports measures including ICC, PCV, MOR, and adjusted odds ratios with 95% confidence intervals. However, the statistical analysis section and presentation should be carefully proofread to ensure that the model specification, model comparison, and interpretation are clear and unambiguous.

Response:
Thank you for this important observation. We carefully reviewed and revised the statistical analysis section to improve the clarity and consistency of the description of the multilevel logistic regression analysis. The revised manuscript clearly describes the two-level structure of the analysis, with children nested within communities, and explains the sequential construction of the null model, individual-level model, community-level model, and final model containing both individual- and community-level factors.

We also clarified the use and interpretation of the intra-class correlation coefficient (ICC), proportional change in variance (PCV), and median odds ratio (MOR), as well as the criteria used for model comparison. Model fit was assessed using deviance, AIC, BIC, and DIC, and the final model was selected based on the reported model-fit parameters.

In addition, we reviewed the reporting of adjusted odds ratios (AORs), 95% confidence intervals, and p-values throughout the Results section and tables to ensure consistency between the text and tabulated findings.

Changes made in the manuscript:
The statistical analysis and model-fitting sections were carefully reviewed and edited, and the presentation of the multilevel regression results was standardized throughout the manuscript.

Comment 2: Data Availability and Ethics

Reviewer comment:
The study is a secondary analysis of anonymized DHS data. The manuscript should clearly

explain the ethical basis of the secondary analysis and should direct readers to the DHS Program for data access. The Data Availability Statement should not imply that the authors can independently redistribute the DHS datasets.

Response:
Thank you for this valuable comment. We agree that the previous wording of the ethics and data availability statements did not adequately distinguish the original KDHS data collection and informed-consent process from the present secondary analysis, and it could also have implied that the authors could independently provide the DHS dataset.

We have therefore substantially revised these statements. The revised manuscript now clearly identifies the study as a secondary analysis of anonymized and de-identified data from the 2022 Kenya Demographic and Health Survey. It states that the data were obtained from the DHS Program following its data-access procedures and that informed consent was obtained during the original KDHS data collection.

We also clarified that no additional informed consent was required for the present analysis because the study involved secondary analysis of de-identified data and did not involve direct contact with participants.

Importantly, the Data Availability Statement now explicitly identifies the DHS dataset as third-party data and states that the authors do not have permission to independently redistribute the datasets. Researchers interested in accessing the data are directed to register with the DHS Program and follow the applicable procedures for obtaining access to the 2022 KDHS dataset.

Changes made in the manuscript:
The sections entitled **“Ethics approval and consent to participate,” “Consent for publication,” and “Availability of data and material”** have been revised accordingly.

Comment 3: Language and Presentation

Reviewer comment:
Language and presentation remain the principal areas requiring attention before publication. There are several grammatical, typographical, and presentation errors, including examples such as “KeyneKenya,” overlapping terminology such as “full immunization measles vaccine coverage,” unresolved alternative wording, and inconsistent numerical formatting. The manuscript should undergo a complete final proofreading rather than addressing only the examples identified by the reviewer.

Response:
Thank you for highlighting this important issue. We agree that careful final proofreading is essential before publication.

We conducted a comprehensive review of the manuscript rather than limiting the revisions to the examples identified in the review. We specifically checked the manuscript for grammatical and typographical errors, duplicated or overlapping wording, incomplete or unresolved alternative phrasing, inconsistent terminology, numerical formatting, punctuation, spacing, and consistency between the Abstract, Methods, Results, Discussion, tables, and Conclusion.

The specific wording and typographical problems identified by the reviewer were reviewed and corrected. We also standardized the terminology used throughout the manuscript, particularly the wording referring to **full immunization coverage among children aged 12–23 months**.

The numerical presentation was also carefully checked. For example, the prevalence estimate is consistently presented as **79.4% (95% CI: 78.13–81.25)** in the Abstract, Results, and Discussion.
We additionally reviewed the tables for consistency in the presentation of AORs, p-values, confidence intervals, reference categories, and decimal places.

The revised manuscript was subsequently proofread in its entirety to remove residual editing text and improve readability and presentation.

Changes made in the manuscript:
The entire manuscript was proofread and edited for grammar, spelling, terminology, sentence construction, numerical formatting, punctuation, and consistency of presentation.

Comment 4: Overall Presentation and Final Editorial Quality

Reviewer comment:
The manuscript is generally understandable, but a final editorial review is required to ensure that no residual tracked changes, editing text, duplicated wording, or formatting inconsistencies remain. The final clean manuscript should be carefully checked before submission.

Response:
Thank you for this recommendation. We have undertaken a final editorial review of the manuscript. We checked the manuscript from the title through the references and supporting information for residual editing text, duplicated wording, inconsistent terminology, grammatical errors, numerical inconsistencies, and formatting problems.

We also reviewed the consistency of the study terminology across the Abstract, Methods, Results, Discussion, Conclusion, tables, and supporting information. The clean version submitted with the revision is intended to contain no tracked changes or unresolved editorial comments.

We appreciate the reviewer's careful attention to the presentation and readability of the manuscript, which has helped us improve the overall quality and clarity of the revised submission.

Final Response

We sincerely thank the reviewer for the constructive comments. We have carefully considered all of the concerns raised and have revised the manuscript accordingly. In particular, we strengthened the explanation of the statistical analysis, clarified the ethics and data-access statements for the secondary analysis of KDHS data, and conducted comprehensive proofreading and editorial revision throughout the manuscript. We believe these revisions have substantially improved the clarity, consistency, and presentation of the manuscript.
