# Supplementary material for: Prevalence and Associated Factors of Full Immunization Coverage among children aged 12–23 months in Kenya: A multi-level logistic regression analysis
Source: PLoS One. 2026 Nov 6;21(11):e0361187. doi: 10.1371/journal.pone.0361187 (PMC13742959; doi:10.1371/journal.pone.0361187)
Supplement: Peer review history

# Peer review: Decision Letter 0

**DOI:** 10.1371/journal.pone.0361187.r001

**Authors:** Edison Mworozi

9 Jul 2026

Dear Dr. Lakew,

Thank you for submitting your manuscript to PLOS ONE. After careful consideration, we feel that it has merit but does not fully meet PLOS ONE’s publication criteria as it currently stands. Therefore, we invite you to submit a revised version of the manuscript that addresses the points raised during the review process. These are highlighted below.

Please submit your revised manuscript by  Aug 23 2026 11:59PM. If you will need more time than this to complete your revisions, please reply to this message or contact the journal office at plosone@plos.org. When you're ready to submit your revision, log on to https://www.editorialmanager.com/pone/ and select the 'Submissions Needing Revision' folder to locate your manuscript file.

- A letter that responds to each point raised by the reviewer(s). You should upload this letter as a separate file labeled 'Response to Reviewers'.
- A marked-up copy of your manuscript that highlights changes made to the original version. You should upload this as a separate file labeled 'Revised Manuscript with Track Changes'.
- An unmarked version of your revised paper without tracked changes. You should upload this as a separate file labeled 'Manuscript'.

If you would like to make changes to your financial disclosure, please include your updated statement in your cover letter. Guidelines for resubmitting your figure files are available below the reviewer comments at the end of this letter.

If applicable, we recommend that you deposit your laboratory protocols in protocols.io to enhance the reproducibility of your results. Protocols.io assigns your protocol its own identifier (DOI) so that it can be cited independently in the future. For instructions see: https://journals.plos.org/plosone/s/submission-guidelines#loc-laboratory-protocols. Additionally, PLOS ONE offers an option for publishing peer-reviewed Lab Protocol articles, which describe protocols hosted on protocols.io. Read more information on sharing protocols at https://plos.org/protocols?utm_medium=editorial-email&utm_source=authorletters&utm_campaign=protocols.

As the corresponding author, your ORCID iD is verified in the submission system and will appear in the published article. PLOS supports the use of ORCID, and we encourage all coauthors to register for an ORCID iD and use it as well. Please encourage your coauthors to verify their ORCID iD within the submission system before final acceptance, as unverified ORCID iDs will not appear in the published article. *Only*  the individual author can complete the verification step; PLOS staff *cannot*  verify ORCID iDs on behalf of authors.

We look forward to receiving your revised manuscript.

Kind regards,

Edison Arwanire Mworozi, M.D

Academic Editor

PLOS One

Journal Requirements:

When submitting your revision, we need you to address these additional requirements.

1. Please ensure that your manuscript meets PLOS ONE's style requirements, including those for file naming. The PLOS ONE style templates can be found at

https://journals.plos.org/plosone/s/file?id=wjVg/PLOSOne_formatting_sample_main_body.pdf and

https://journals.plos.org/plosone/s/file?id=ba62/PLOSOne_formatting_sample_title_authors_affiliations.pdf

2. Please note that your Data Availability Statement is currently missing the accession number of each dataset or a direct link to access each database. If your manuscript is accepted for publication, you will be asked to provide these details on a very short timeline. We therefore suggest that you provide this information now, though we will not hold up the peer review process if you are unable.

3.  Please include captions for your Supporting Information files at the end of your manuscript, and update any in-text citations to match accordingly. Please see our Supporting Information guidelines for more information: http://journals.plos.org/plosone/s/supporting-information.

Reviewers' comments:

Reviewer's Responses to Questions

**Comments to the Author**

1. Is the manuscript technically sound, and do the data support the conclusions?

Reviewer #1: No

Reviewer #2: Partly

Reviewer #3: No

Reviewer #4: Partly

**********

2. Has the statistical analysis been performed appropriately and rigorously?

Reviewer #1: No

Reviewer #2: I Don't Know

Reviewer #3: No

Reviewer #4: No

**********

3. Have the authors made all data underlying the findings in their manuscript fully available?

The PLOS Data policy

Reviewer #1: Yes

Reviewer #2: No

Reviewer #3: No

Reviewer #4: No

**********

4. Is the manuscript presented in an intelligible fashion and written in standard English?

Reviewer #1: Yes

Reviewer #2: No

Reviewer #3: No

Reviewer #4: No

**********

Reviewer #1: This manuscript has serious methodological flaws, and thus requires major revisions.

The main problem is that the outcome (or response) variable studies was the vaccination status of 0-5 year old children but the 0-1 year old among them would not have had the opportunity to complete the infant immunization schedule until they were 11 month 29 days old. This would necessarily lower the proportion of vaccinated children. Therefore the entire analysis needs to be redone after correcting for this major flaw.

Some specific issues.

L68-9: Please specify the "recommended vaccines" by first year of life because the DHS report uses two benchmarks - "basic antigens" and the "national schedule".

L84-89: Summary comments across several studies. Should be moved to Discussion section.

L117-119: Should be deleted as this study does not assess anything about governance structure etc.

L130: Questions about vaccination might have been asked about under-five children. But DHS 2022 published vaccination proportions for only 12-23 month old children. It is difficult to understand why the authors included the 0-1 year age group also as they will not have reached the age when vaccination would be completed.

L133: Weighting - what were the weighting principles followed? Was it just like DHS 2022? Please state that clearly and provide further details.

L149-150: It is not clear how the authors classified the community on educational attainment and poverty. Even the tables are quite vague and potentially subjective as they lack numerical details.

L170 and other places: The authors state "greater proportion" of mothers but they do not give any numbers or percent. They need to specify the cut-off proportions.

L176: "sanitize the data" - what does this mean? If the authors meant data cleaning, they need to describe precisely what parameters they used to query and clean the DHS 2022 data.

L181: "unique technique" - This is a well known technique. Why are the authors calling it unique?

Table 1: The age grouping mixes up infants with 1-2 year old children. Almost 22% of the sample (0-11 month old infants) would not have had the chance to complete infant immunization schedule.

Table 1: Household's wealth status is described very subjectively - Was the partitioning by quintiles?

Table 1: Sex categorization is missing.

Table 1: Size of child - was this size or weight at birth? In absence of numbers or clear criteria, the analytical framework becomes fuzzy.

Reviewer #2: Thank you for the opportunity to review this manuscript. The study addresses an important public health topic, full immunization coverage among children in Kenya and individual- and community-level factors associated with vaccination status. The study uses a large, nationally representative dataset and has the potential to make a useful contribution if the authors strengthen the methods and reporting.

I do have some concerns about the current version (namely methodological and reporting) that lower confidence in the validity and interpretation of the study findings. I am recommending major revision. The manuscript may be suitable for PLOS once the authors successfully address the highlighted issues.

MAJOR/MODERATE

• Clear definition for “full immunization”: In the manuscript, the study population is described as children under 5 but full immunization is defined as having “received all recommended vaccines by the age of 12–23 months.” The sample includes children aged 0–59 months, with children both below 12-23 month (0-6 months and 7-12 months, who are seemingly not eligible to be considered fully immunized) and above (25-59 months). As a result, it is not clear how the authors determined full immunization status for all the different ages. It appears the analysis included all under-five children and not just children 12-23 months. Since children younger than 12 months (can’t meet the study definition of fully immunized) and older than 23 months were included, it would be helpful for the authors to justify why they chose this age range and explain how they handled vaccination eligibility as well as timing. Although the authors already included a basic definition of full immunization, it would be helpful to provide a clear operational definition of full immunization that includes which vaccines/doses were included and how they determined vaccination status (i.e., vaccination cards, maternal/caregiver recall, both).

• Clarify sample weighting: The methods section mentions that weighted frequencies were used and that the data were weighted, but there are not enough details on how sampling weights, clustering, and stratification were incorporated into the regression analyses. The methods should clearly describe the weights used, whether/how weights were applied in the multilevel models, how the authors accounted for clustering/stratification as well as name the software used for analyses.

• Clarify model specification and selection: There are a few potential concerns with the multilevel model:

o The authors note that the “To identify the best-fitting model, we looked at different criteria: Likelihood Ratio test (LLR), deviance, Akaike information criterion (AIC), and deviance information criterion (DIC). The model with the lowest value for one of these four criteria was chosen as the most accurate representation of the data”. It is not clear how the authors actually selected the best fit model, especially since when they write about model fitness parameters, it does not seem consistent with what is included in the methods section, e.g. log-likelihood ratio was highest in Model III, “indicating better model likelihood.” I suggest clarifying model selection.

o The authors state that variables with p-value <0.25 were considered for multivariable analysis but do not provide a rationale. Recommending that the authors provide justification for choosing this threshold and clarify whether variable selection was only based on statistical significance.

o In Table 2, the MOR values reported for three of the models are below 1. Since I believe MOR cannot be less than 1, the reported values suggest a possible error (calculation, reporting, etc.). Please review and recalculate, and revise the interpretation if needed.

• Fix outcome inconsistencies: The manuscript mentions measles vaccine coverage a few times even though the study outcome is full immunization coverage. Please carefully revie w and remove or correct mention of measles vaccine coverage if the study did not analyze measles vaccine coverage.

o ”…the study can provide a more comprehensive picture of what contributes to childhood measles coverage…”

o ”The intraclass correlation coefficient (ICC) indicated what proportion of the overall difference in measles vaccine coverage…”

o ”…to examine the presence of clustering and heterogeneity of measles vaccine coverage…”

• Improve operational definitions: The authors refer to communities with “higher” or “greater” proportions of certain characteristics, but the exact thresholds they used, and method to categorize are not always clear. The operational definition for community-level education could use some more clarity. For example, the authors write “a greater proportion of mothers or caregivers” and it is not clear what “greater than” refers to (greater than what?). Please specify how these variables were constructed, what cut points/thresholds were used, whether weighted proportions were used, and any other relevant details so that someone could reasonably reproduce this. Additionally, more information should be given for the description of birth size, including that it refers to “perceived size at birth” and how it was measured in the study (maternal/caregiver recall?).

• Use caution with interpretations: Since the study is cross-sectional, the authors should avoid using causal language, such as “leads to improved” or “influence.” Some statements seem to imply that interventions targeting specific factors would improve immunization coverage. These should be revised to make it clear that the study findings show associations, not causal effects.

• Review and correct citations: Multiple references do not support claims they are supposed to backup. Please carefully check all references to make sure that they directly support the corresponding statements. If a source supports a broad point or general concept, the language should be softened/tweaked to reflect the evidence presented in that source or otherwise replaced with a more appropriate reference.

Examples include:

o Ref 5: this reference does not seem to support the description of KEPI or the vaccination target by one year of age. Please verify and replace with a more appropriate source, such as WHO or Kenya Ministry of Health.

o Ref 13: this reference does report higher immunization coverage among children whose mothers attended ANC in the descriptive analyses but not in the multivariable model. Suggest revising the wording/softening the wording to reflect the nature and strength of the evidence.

o Ref 14: this reference may not directly support the claim about ANC visits, since it does not seem like the study looked at this specifically (though does support that counseling is important).

o Ref 15: this reference does not seem to look at ANC visits as a predictor of full immunization, nor does it discuss ANC as a way to provide vaccination counselling to parents. The reference does identify several maternal and socioeconomic determinants of immunization coverage, it doesn’t provide direct support for the statement about ANC. Suggest replacing with a citation that specifically evaluates ANC use or revising the text to more broadly reflect maternal health factors.

o Ref 19: this reference backs up the general idea that maternal characteristics can be associated with child vaccination outcomes. However, it doesn't support the specific claim about older mothers’ experience, autonomy, and health information. Suggest either tweaking the sentence a little or finding another, more appropriate source.

o Ref 20: this reference is not an appropriate source to support claims about older mothers since the study does not look at maternal age.

o Ref 12: this reference looks at HBV vaccination uptake among healthcare students and it does not appear to address parity or maternal barriers to childhood immunization. Recommend the authors either remove or replace this source since it does not seem to support this sentence.

• Make sure discussion section closely aligns with the results: In some areas of the discussion section, it is not always clear what is a finding from this study and what comes from the published literature. As well, some interpretations seem a little speculative (e.g. caregiver perceptions of birth size, outreach programs in rural areas, competing demands in larger families). These should be framed more as possible explanations or hypotheses, rather than as established conclusions. As well, the authors should consider adding potential misclassification of immunization status to the study limitations.

• Improve consistency and statistical reporting: Recommend using consistent statistical terms throughout the manuscript. For instance, the authors use AOR in the abstract and report ORs in the results section.

The p-values reported as 0.000 should be reported as p < 0.001 instead. Percentages and weighted frequencies should be checked for consistency throughout the manuscript. For example, in the text, the authors report that “Most research participants, 10,894 people, or 62.81%, came from rural areas” whereas the percent of rural participants (n= 10,894.77) is reported as 62.61% in Table. Please check and verify all values or provide explanations for variation.

MINOR

• The manuscript would benefit from some careful editing to improve readability. For example, “sanitize” could be replaced with “clean,” and “incapacity” could be replaced with “inability”. Similarly, words like “lucid” and “closeness in traits” could also be replaced with words used more conventionally in this context.

In some areas, the wording used is too strong and could be toned done a little bit, for example "guaranteed" and “unique”.

• Please make sure terminology is used consistently (for example, “full immunization,” “full vaccination,” and “full vaccine coverage.”).

• Table formatting could be improved. Some headings are unclear or split awkwardly across lines, and capitalization is inconsistent. Consider including a heading for individual-level factors (like is included for community-level factors).

Reviewer #3: This study uses the 2022 KDHS data to examine factors associated with full immunization coverage among children in Kenya. The topic is relevant and the use of multilevel modelling is a commendable approach. However, the manuscript contains fatal methodological errors and statistical inconsistencies that render the reported findings invalid. These issues cannot be addressed through minor revisions; the analysis must be fundamentally restructured.

Major Methodological Flaws

1. Incorrect target population for the outcome variable

The authors include children aged 0–6 months and 7–12 months in the full sample (Table 1), yet define full immunization as “receiving all recommended vaccines by 12–23 months of age” (lines 141–143). Children under 12 months are biologically ineligible to have completed the full schedule. Coding them as “not fully immunized” introduces severe survival/time‑related bias and endogeneity. With an incorrect denominator, the reported coverage estimate (68.8%) and all adjusted odds ratios are invalid.

2. Contradictory random-effect parameters in the multilevel model

In Table 2, the community‑level variance in the null model is 0.13, yet in the final model (Model III) it more than doubles to 0.27. Simultaneously, the authors report a positive proportional change in variance (PCV) of 11.3% (i.e., a decrease). Statistically, adding explanatory variables should reduce the unexplained between‑cluster variance. An increased variance alongside a claimed reduction indicates either model nonconvergence or serious misreporting of parameters, making the ICC and MOR estimates unreliable.

3. Improper handling of key independent variables

Antenatal care (ANC): The “Yes” category accounts for 98.88% (line 233). Such extreme class imbalance requires penalized estimation or recategorization; otherwise, odds ratios are highly unstable.

Birth size: The original three categories (Large/Average/Small) appear in Table 1, but the final model (Table 3) only reports “Average” versus “Large”. The “Small” category is omitted without any justification.

Language and Academic Writing Issues

1.Critical terminology errors: “Kenya” is misspelled as “Keyna/Keyne” (lines 121,125，204). These errors suggest insufficient proofreading.

2.Inappropriate causal language: Given the cross‑sectional design, the authors should use “associated factors” rather than “determinants” or “influenced by” throughout the abstract and discussion.

3.Inconsistent hierarchical framework: The methods describe five administrative levels (lines 102–119), yet the statistical model uses only a basic two‑level structure (individual + community). This disconnect between conceptual framework and model specification is not addressed, and the handling of DHS sampling weights is not explained.

Reviewer #4: My general comments to the authors:

This manuscript examines factors associated with full immunization coverage in Kenya using KDHS 2022 data. The topic is relevant and the use of a nationally representative survey and multilevel analysis could make a useful contribution. However, major revisions are needed before the findings can be interpreted as estimates of full immunization coverage in Kenya or used to inform National Vaccines and Immunization Programme (NVIP)

programming.

Title:

The title should be aligned with the outcome actually measured. It currently refers to 'full immunization coverage among under-five children.' In the manuscript, full immunization is defined as completion of recommended vaccines by 12–23 months yet the analysis includes children aged 0–59 months. The title should either specify the correct FIC age group or use a different outcome label that accurately reflects an under-five analysis.

Background:

The Background provides a useful rationale for examining inequities in immunization uptake. However, the national programme is referred to as the ‘Kenya Expanded Programme on Immunization (KEPI).’ This should be updated throughout to the National Vaccines and Immunization Programme (NVIP).

The authors should also accurately define Kenya’s FIC indicator. In Kenya, this includes completion of the relevant infant antigens including MR1 and at least one dose of Vitamin A supplementation at 6 months. The manuscript currently does not state the antigen schedule or Vitamin A component. Claims that this is the ‘first national research’ using a more comprehensive approach should be supported with a systematic literature review or removed.

Abstract:

The abstract reports 68.8% ‘fully vaccinated’ among under-five children but does not define full immunization coverage. It should state the age group, the exact vaccine schedule used, whether Vitamin A was included and whether vaccination history came from cards, maternal recall or both. Kenya’s KDHS 2022 distinguishes coverage for basic antigens from coverage according to the national schedule, these should not be mixed up. The KDHS reports 80% coverage for basic antigens among children aged 12–23 months and 55% fully vaccinated according to the national schedule underscoring why the manuscript must precisely define its outcome.

Methods:

(i) Data source and study population

The study appropriately identifies KDHS 2022 as its analytic data source. The Kenya Population and Housing Census should be described only as the basis for the sampling frame not as a separate source of study data.

The central methodological concern is the denominator. The manuscript uses a weighted sample of 17,401 children aged 0–59 months while defining full immunization in relation to children aged 12–23 months. Children younger than 12 months may not yet be eligible for all routine doses while children aged 24–59 months are outside the standard age group used for reporting full immunization coverage. The authors should restrict the primary analysis to the relevant age group and report the resulting weighted and unweighted sample sizes.

(ii) Definition of full immunization coverage:

The outcome definition requires substantial revision. The manuscript defines full immunization only as receipt of ‘all recommended vaccines’ without specifying the included doses, antigens or related interventions.

The authors should explicitly state the definition used, list all included doses, identify the exact KDHS variables used and provide a reproducible coding algorithm. If the intended outcome is Kenya’s full immunization coverage indicator, the definition should include completion of the relevant infant schedule including MR1 and at least one Vitamin A dose from six months as applicable to the national indicator. If the KDHS dataset does not permit the exact national definition, the authors should use a more accurate label, such as ‘vaccination coverage according to the KDHS national schedule’ and acknowledge the difference.

Statistical analysis:

Multilevel logistic regression is conceptually suitable because KDHS observations are clustered within enumeration areas. However, the methods are insufficiently detailed to establish that the analysis was conducted rigorously. The authors should state how survey weights, stratification and clustering were incorporated, how missing data were handled, whether multicollinearity was assessed, how independent variables were coded and why p<0.25 was used for selection into the multivariable model.

The manuscript also shifts between ‘full immunization,’ ‘full vaccination’ and ‘measles coverage.’ These are different measures and must not be used interchangeably. References to measles coverage in the analytic methods section should be corrected if the intended outcome is full immunization coverage.

The model fit statistics and their interpretation require verification especially the reported median odds ratios, confidence intervals and the relationship between the reported community level variance and ICC.

Results:

The reported 68.8% estimate cannot currently be interpreted as Kenya’s full immunization coverage because the denominator includes all under-five children rather than the relevant FIC age group. The authors should rerun the analysis after correcting the study population and outcome definition then update the abstract, tables, results narrative and discussion.

Tables should be revised for clarity. Reference categories, weighted denominators, category labels and missing-data handling should be clearly shown. The outcome terminology should be standardized throughout as full immunization coverage/status.

Discussion:

The observed associations with maternal education, ANC attendance, facility delivery, household composition and media exposure are plausible and relevant to immunization service use in Kenya. However, the cross-sectional design supports association not causation. The authors should avoid wording that implies these factors caused higher full immunization coverage.

The discussion should not attribute higher rural coverage to successful outreach programmes unless the study contains evidence for that explanation. It should instead present this as a hypothesis and consider alternative explanations including differences in service use, survey measurement or the outcome definition.

The limitations should address vaccination card availability and recall bias, absence of timeliness analysis, residual confounding, potential outcome misclassification and the distinction between KDHS survey estimates and routine administrative reporting. This study uses KDHS data it does not analyse routine data from KHIS/DHIS2.

Conclusion:

The conclusion should be revised after the full-immunization outcome and age group are corrected. It should state that the study identifies factors associated with full immunization coverage rather than implying that the identified factors or proposed interventions will directly improve coverage.

Ethics and data availability:

The ethics text should be consolidated as substantially similar statements appear more than once. The manuscript should clearly state that it used anonymized secondary KDHS data accessed through the DHS Program.

The Data Availability Statement should provide the precise DHS access pathway and clarify that raw KDHS microdata are third-party data subject to DHS access terms. The authors could also provide supporting information such as variable-recoding guide and full-immunization outcome (just but a proposal)

Language and presentation:

The manuscript needs comprehensive English language editing. It contains grammatical errors, incomplete sentences, informal wording, typographical errors and inconsistent terminology.

For examples: ‘whether their child had experienced taken full immunizations,’ 'Excel was used to sanitize the data’ and inconsistent references to Kenya.

In summary, the manuscript has a potentially valuable research question and dataset, but it requires major revision.

The priority is to define and reconstruct full immunization coverage accurately, align the denominator with the intended indicator, transparently document the analysis and then revise the results, interpretation, terminology and language accordingly.

**********

what does this mean?). If published, this will include your full peer review and any attached files.

If you choose “no”, your identity will remain anonymous but your review may still be made public.

**Do you want your identity to be public for this peer review?**  For information about this choice, including consent withdrawal, please see our Privacy Policy

Reviewer #1: No

Reviewer #2: No

Reviewer #3: No

Reviewer #4: **Yes:**  Adungosi Jeniffer Namazzi

**********

[NOTE: If reviewer comments were submitted as an attachment file, they will be attached to this email and accessible via the submission site. Please log into your account, locate the manuscript record, and check for the action link "View Attachments". If this link does not appear, there are no attachment files.]

To ensure your figures meet our technical requirements, please review our figure guidelines: https://journals.plos.org/plosone/s/figures

You may also use PLOS’s free figure tool, NAAS, to help you prepare publication quality figures: https://journals.plos.org/plosone/s/figures#loc-tools-for-figure-preparation.

NAAS will assess whether your figures meet our technical requirements by comparing each figure against our figure specifications.

# Author comment: Author response to Decision Letter 1

**DOI:** 10.1371/journal.pone.0361187.r002

14 Jul 2026

Thank you for your valuable comments. The responses to the editor and reviewer comments are attached

# Peer review: Decision Letter 1

**DOI:** 10.1371/journal.pone.0361187.r003

**Authors:** Edison Mworozi

25 Sep 2026

Dear Dr. Lakew,

Thank you for submitting your manuscript to PLOS One. After careful consideration, we feel that it has merit but does not fully meet PLOS One’s publication criteria as it currently stands. Therefore, we invite you to submit a revised version of the manuscript that addresses the points raised during the review process.

Please submit your revised manuscript by Nov 09 2026 11:59PM. If you will need more time than this to complete your revisions, please reply to this message or contact the journal office at plosone@plos.org. When you're ready to submit your revision, log on to https://www.editorialmanager.com/pone/ and select the 'Submissions Needing Revision' folder to locate your manuscript file.

- A letter that responds to each point raised by the academic editor and reviewer(s). You should upload this letter as a separate file labeled 'Response to Reviewers'.
- A marked-up copy of your manuscript that highlights changes made to the original version. You should upload this as a separate file labeled 'Revised Manuscript with Track Changes'.
- An unmarked version of your revised paper without tracked changes. You should upload this as a separate file labeled 'Manuscript'.

If you would like to make changes to your financial disclosure, please include your updated statement in your cover letter. Guidelines for resubmitting your figure files are available below the reviewer comments at the end of this letter.

If applicable, we recommend that you deposit your laboratory protocols in protocols.io to enhance the reproducibility of your results. Protocols.io assigns your protocol its own identifier (DOI) so that it can be cited independently in the future. For instructions see: https://journals.plos.org/plosone/s/submission-guidelines#loc-laboratory-protocols. Additionally, PLOS One offers an option for publishing peer-reviewed Lab Protocol articles, which describe protocols hosted on protocols.io. Read more information on sharing protocols at https://plos.org/protocols?utm_medium=editorial-email&utm_source=authorletters&utm_campaign=protocols.

As the corresponding author, your ORCID iD is verified in the submission system and will appear in the published article. PLOS supports the use of ORCID, and we encourage all coauthors to register for an ORCID iD and use it as well. Please encourage your coauthors to verify their ORCID iD within the submission system before final acceptance, as unverified ORCID iDs will not appear in the published article. *Only*  the individual author can complete the verification step; PLOS staff *cannot*  verify ORCID iDs on behalf of authors.

We look forward to receiving your revised manuscript.

Kind regards,

Jennifer Tucker, PhD, Senior Editor, PLOS Editorial Office on behalf of

Edison Arwanire Mworozi, M.D

Academic Editor

PLOS One

Journal Requirements:

If the reviewer comments include a recommendation to cite specific previously published works, please review and evaluate these publications to determine whether they are relevant and should be cited. There is no requirement to cite these works unless the editor has indicated otherwise.

Please review your reference list to ensure that it is complete and correct. If you have cited papers that have been retracted, please include the rationale for doing so in the manuscript text, or remove these references and replace them with relevant current references. Any changes to the reference list should be mentioned in the rebuttal letter that accompanies your revised manuscript. If you need to cite a retracted article, indicate the article’s retracted status in the References list and also include a citation and full reference for the retraction notice.

Additional Editor Comments:

Please address reviewer 2 comments regarding language and structure.

[Note: HTML markup is below. Please do not edit.]

Reviewers' comments:

Reviewer's Responses to Questions

**Comments to the Author**

Reviewer #2: All comments have been addressed

Reviewer #4: All comments have been addressed

**********

2. Is the manuscript technically sound, and do the data support the conclusions?

Reviewer #2: Yes

Reviewer #4: Yes

**********

3. Has the statistical analysis been performed appropriately and rigorously?

Reviewer #2: I Don't Know

Reviewer #4: Yes

**********

4. Have the authors made all data underlying the findings in their manuscript fully available?

The PLOS Data policy

Reviewer #2: Yes

Reviewer #4: Yes

**********

5. Is the manuscript presented in an intelligible fashion and written in standard English?

Reviewer #2: Yes

Reviewer #4: No

**********

Reviewer #2: The authors have substantially improved the manuscript with each round of review and have addressed my previous concerns/comments. I am satisfied and support publication of this manuscript. As minor points, the authors may want to: 1) verify the 95% CI reported for the small birth size category in Table 3; 2) check the accuracy of Reference 27 ("Physiology of lipid digestion and absorption in poultry...") since it does not seem relevant; and 3) double check formatting consistency throughout (e.g., consistent line spacing and consistent use of sentence, case especially after colons).

Reviewer #4: OVERALL ASSESSMENT

Thank you for the opportunity to review the revised manuscript. The authors have made important efforts to strengthen the manuscript and address the substantive issues raised during the previous review. Overall, the study addresses a relevant public health question and provides useful evidence on individual and community level factors associated with full immunization among children aged 12–23 months in Kenya. The use of nationally representative KDHS 2022 data and a multilevel analytical approach are important strengths. However, the revised manuscript still requires a thorough final editorial and language review before publication, particularly to remove residual editing/tracked change text and correct grammatical and typographical errors.

ABSTRACT

The abstract generally provides an appropriate summary of the study objective, methods, principal findings and conclusions. The focus on both individual and community-level determinants is clear and consistent with the analytical approach. However, several language and formatting issues remain. For example, expressions such as ‘children aged from 12 to 23 months’ should consistently read ‘children aged 12–23 months.’ Numerical reporting and confidence intervals should also be checked carefully for consistent formatting.

INTRODUCTION

The introduction provides an appropriate rationale for examining full immunization coverage in Kenya and recognizes that vaccination uptake may be influenced by factors operating at both individual and community levels. The rationale for using a multilevel approach is therefore appropriate. Also, the terminology for Kenya’s national immunization programme should be made consistent. On page 34 (lines 71–74), both ‘Expanded Programme on Immunization (KEPI)’ and ‘National Vaccines and Immunization Programme (NVIP)’ appear together. The residual KEPI wording should be removed and National Vaccines and Immunization Programme (NVIP) used consistently. I recommend a final review for conciseness, grammatical clarity and consistency of terminology throughout this section.

METHODS

The methodological approach is generally appropriate for the research question. The study uses nationally representative KDHS 2022 data and applies a two level binary logistic regression model to account for the nested nature of children within communities. The revised manuscript also describes the use of sampling weights and consideration of the survey sampling structure.

The definition of full immunization and the analytical approach are sufficiently described to understand how the outcome and associated factors were assessed. The use of adjusted odds ratios and 95% confidence intervals is appropriate. However, the section requires careful proofreading because some sentences contain apparent remnants of revisions. For example, the phrase ‘to explore factors linked identify factors associated with to full immunization coverage’ is clearly an unresolved editing error. Such errors should be corrected throughout the final manuscript.

RESULTS

The results generally correspond with the stated objectives and analytical methods and the presentation of adjusted associations enables interpretation of the individual and community-level factors associated with full immunization.

Nevertheless, numerical and formatting consistency should be checked throughout the tables and narrative. For example, the reported prevalence appears as ‘% 79.4%’ and the confidence interval is presented as ‘78.13 81.25,’ which requires correction. All percentages, confidence intervals, sample sizes, table headings and statistical measures should therefore undergo a final verification against the analytical outputs.

DISCUSSION

The discussion generally interprets the findings within the context of immunization uptake and appropriately considers factors such as antenatal care, institutional delivery, maternal education, media exposure, household characteristics and place of residence.

Importantly, the revised manuscript acknowledges that the cross sectional design does not permit causal inference. It also recognizes potential recall bias where caregiver reports were used, inability to assess vaccination timeliness, residual confounding, and the distinction between KDHS survey estimates and routine KHIS/DHIS2 administrative coverage estimates. These are important limitations and strengthen the interpretation of the findings.

The authors should nevertheless ensure that statements proposing programme or policy responses remain proportionate to the observational nature of the evidence and are framed as implications or considerations rather than demonstrated effects.

CONCLUSION

The conclusion is broadly supported by the findings and appropriately identifies antenatal care, place of delivery, maternal education and household characteristics among the factors associated with full immunization. It also explicitly recognizes the limitations of the cross sectional design and the need for further research to better understand barriers to immunization.

I recommend maintaining this cautious language and avoiding any wording that could imply causality.

STATISTICAL ANALYSIS

The statistical approach is appropriate overall. The multilevel logistic regression framework is suitable for the hierarchical structure of DHS data and for examining individual and community level factors. The manuscript describes assessment of between community variation using ICC, PCV and MOR and reports adjusted odds ratios with 95% confidence intervals.

However, the statistical analysis section contains residual editing and language errors that should be corrected to ensure that the final description of model specification, model comparison and interpretation is unambiguous.

DATA AVAILABILITY AND ETHICS

The manuscript states that this is a secondary analysis of anonymized DHS data obtained following permission from the DHS Program. It also explains that informed consent was obtained as part of the original DHS data collection and that the secondary analysis used de-identified observations. This is appropriate for the nature of the study.

The final Data Availability Statement should clearly direct readers to the DHS Program and explain any applicable registration or access requirements rather than suggesting that the authors themselves can freely redistribute the third-party dataset.

I identified no specific concerns regarding dual publication, research ethics or publication ethics from the materials reviewed.

LANGUAGE AND PRESENTATION

This remains the principal issue requiring attention before publication. Although the manuscript is generally understandable, there are multiple grammatical, typographical and residual editing errors. Examples include ‘KeyneKenya,’ overlapping terminology such as ‘full immunization measles vaccine coverage’ and sentences containing alternative wording that appears not to have been resolved during revision.

Given that PLOS ONE does not copyedit accepted manuscripts, I strongly recommend that the authors undertake a complete final proofreading of the clean manuscript rather than correcting only the examples identified by the reviewer. Particular attention should be given to grammar, sentence construction, duplicated words, residual tracked changes, terminology, numerical formatting and consistency across the abstract, main text, tables and conclusions.

OVERALL RECOMMENDATION

The substantive scientific aspects of the manuscript have been considerably strengthened and the data and analytical approach generally support the principal conclusions. The remaining concerns are predominantly related to language, presentation and final editorial quality rather than the fundamental scientific validity of the study. I therefore encourage the authors to undertake a comprehensive final language and formatting review before the manuscript proceeds to publication.

**********

what does this mean?). If published, this will include your full peer review and any attached files.

If you choose “no”, your identity will remain anonymous but your review may still be made public.

**Do you want your identity to be public for this peer review?**  For information about this choice, including consent withdrawal, please see our Privacy Policy

Reviewer #2: No

Reviewer #4: **Yes:**  Jeniffer Namazzi Adungosi

**********

[NOTE: If reviewer comments were submitted as an attachment file, they will be attached to this email and accessible via the submission site. Please log into your account, locate the manuscript record, and check for the action link "View Attachments". If this link does not appear, there are no attachment files.]

To ensure your figures meet our technical requirements, please review our figure guidelines: https://journals.plos.org/plosone/s/figures

You may also use PLOS’s free figure tool, NAAS, to help you prepare publication quality figures: https://journals.plos.org/plosone/s/figures#loc-tools-for-figure-preparation.

NAAS will assess whether your figures meet our technical requirements by comparing each figure against our figure specifications.

# Author comment: Author response to Decision Letter 2

**DOI:** 10.1371/journal.pone.0361187.r004

1 Oct 2026

Thank you very much for the constructive comments. I have attached the PDF point-by-point response.

Attachment Submitted filename: Response to Reviewers.pdf

# Editor report: Decision Letter 2

**DOI:** 10.1371/journal.pone.0361187.r005

**Authors:** Edison Mworozi

7 Oct 2026

Prevalence and Associated Factors of Full Immunization Coverage among children aged 12–23 months in Kenya:

A multi-level logistic regression analysis

PONE-D-25-21170R2

Dear Gerbrew,

We’re pleased to inform you that your manuscript has been judged scientifically suitable for publication and will be formally accepted for publication once it meets all outstanding technical requirements.

Within one week, you’ll receive an e-mail detailing the required amendments. When these have been addressed, you’ll receive a formal acceptance letter and your manuscript will be scheduled for publication.

An invoice will be generated when your article is formally accepted. Please note, if your institution has a publishing partnership with PLOS and your article meets the relevant criteria, all or part of your publication costs will be covered. Please make sure your user information is up-to-date by logging into Editorial Manager at Editorial Manager® and clicking the ‘Update My Information' link at the top of the page. For questions related to billing, please contact billing support.

If your institution or institutions have a press office, please notify them about your upcoming paper to help maximize its impact. If they’ll be preparing press materials, please inform our press team as soon as possible -- no later than 48 hours after receiving the formal acceptance. Your manuscript will remain under strict press embargo until 2 pm Eastern Time on the date of publication. For more information, please contact onepress@plos.org.

Kind regards,

Edison Arwanire Mworozi, M.D

Academic Editor

PLOS One

Reviewers' comments:

# Editor report: Acceptance letter

**DOI:** 10.1371/journal.pone.0361187.r006

**Authors:** Edison Mworozi

PONE-D-25-21170R2

PLOS One

Dear Dr. Lakew,

I'm pleased to inform you that your manuscript has been deemed suitable for publication in PLOS One. Congratulations! Your manuscript is now being handed over to our production team.

At this stage, our production department will prepare your paper for publication. This includes ensuring the following:

* All references, tables, and figures are properly cited

* All relevant supporting information is included in the manuscript submission,

* There are no issues that prevent the paper from being properly typeset

You will receive further instructions from the production team, including instructions on how to review your proof when it is ready. Please keep in mind that we are working through a large volume of accepted articles, so please give us a few days to review your paper and let you know the next and final steps.

Lastly, if your institution or institutions have a press office, please let them know about your upcoming paper now to help maximize its impact. If they'll be preparing press materials, please inform our press team within the next 48 hours. Your manuscript will remain under strict press embargo until 2 pm Eastern Time on the date of publication. For more information, please contact onepress@plos.org.

You will receive an invoice from PLOS for your publication fee after your manuscript has reached the completed accept phase. If you receive an email requesting payment before acceptance or for any other service, this may be a phishing scheme. Learn how to identify phishing emails and protect your accounts at https://explore.plos.org/phishing.

If we can help with anything else, please email us at customercare@plos.org.

Thank you for submitting your work to PLOS One and supporting open access.

Kind regards,

PLOS One Editorial Office Staff

on behalf of

Professor Edison Arwanire Mworozi

Academic Editor

PLOS One
